# Why not try harder to prove that a text message alert system for trained volunteers saves lives after cardiac arrest?

**DOI:** 10.1007/s12471-023-01786-z

**Published:** 2023-05-09

**Authors:** Paul Calle

**Affiliations:** 1 Emergency Department, General Hospital Maria Middelares, Ghent, Belgium; 2 Faculty of Medicine and Health Sciences, Ghent University, Ghent, Belgium

Dear Editor,

The original article by Oosterveer et al. entitled ‘Improved ROSC rates in out-of-hospital cardiac arrest patients after introduction of a text message alert system for trained volunteers’, published in the January 2023 issue, and the subsequent letter to the editor by Stieglis et al. remind me of two other articles related to public access defibrillation that have previously appeared in the *Netherlands Heart Journal* [1, 2]. Like Stieglis et al., I had several methodological concerns, thereby expressing doubt about the validity of the authors’ conclusions [3, 4].

Being aware that it is impossible to solve all the problems related to a historical control group, I would like to propose—once more—an alternative way to look at the registered data in order to estimate the impact of the intervention under investigation on survival. As survival (preferably neurologically intact survival) is the only parameter that matters, the investigators should focus on this group. How many survivors were reached by the text message (TM) responders before other caregivers? How often were the TM responders the first to bring an automated external defibrillator (AED) to the scene of these surviving patients, and how often did they deliver an AED shock, leading to the return of spontaneous circulation (ROSC)? From the article by Oosterveer et al. we only know that 42 patients (15.9% of all cardiac arrests cases) were reached by the TM responders before the first responders or the ambulance, and that in 31 of these 42 cases an AED was attached. We have no idea how many of these 42 patients survived. Neither is it documented how often the TM responders delivered a (successful) AED shock. In a well-designed study, one should also record the duration of the activities of the TM responders before the arrival of other caregivers. Indeed, ROSC obtained by TM responders does not necessarily mean that the patient would die if there were no TM alert system. Quantification of the surplus value of the TM alert system in a particular patient is difficult. As stated by the European Resuscitation Council [5], one may assume that each minute of defibrillation delay decreases the chances of successful resuscitation by about 3–5%. Finally, the age and co-morbidities of the survivors should also be included.

By combining all these data, an estimation of the quality-adjusted life years saved by the TM alert system during a 1-year period in a region with approximately 775,000 inhabitants can be made. This absolute figure will tell us more than differences in ROSC and survival rates between the study group and the historical control group.
